# The effectiveness of Orem's self-care program on headache-related disability in migraine patients

**Published:** 2016-10-07

**Authors:** Fatemeh Mahmoudzadeh-Zarandi, Farahrooz Hamedanizadeh, Abbas Ebadi, Afsaneh Raiesifar

**Affiliations:** 1 Department of Operation Room, School of Paramedicine, Birjand University of Medical Sciences, Birjand, Iran; 2 Department of Medical Surgical Nursing, School of Nursing, Baqiyatallah University of Medical Sciences, Tehran, Iran; 3 Behavioral Sciences Research Center, Department of Nursing, School of Nursing, Baqiyatallah University of Medical Sciences, Tehran, Iran; 4 Department of Medical Surgical Nursing, School of Nursing and Midwifery, Tehran University of Medical Sciences, Tehran, Iran

**Keywords:** Migraine Headache, Orem Self-care Model, MIDAS, Nursing, Iran

## Abstract

**Background:** Providing a self-care program appropriate for patient needs in a supportive educative nursing system format could reduce migraine-induced disability. This study was designed to determine the effectiveness of Orem's self-care program on headache related disability in migraine patients.

**Methods:** In this randomized clinical trial, episodic migraine patients with or without aura who signed the informed consent were randomly assigned to two groups (44 patients each). The data collection tools included a demographic questionnaire, the Migraine Disability Assessment (MIDAS) questionnaire, an Orem cognition form, and a self-care checklist. The programs were held as four 30 to 45 minutes training sessions for experimental group. The MIDAS were filled out before and three months after program in two groups. Data were analyzed with SPSS statistical software, version 16 and using chi-square, Mann-Whitney and Wilcoxon tests.

**Results:** There was no statistically significant difference between the two groups in terms of demographic variables (P > 0.05). The mean total MIDAS score in the experimental group, before and after the intervention was 28.1 ± 17.5 and 6.03 ± 4.52, respectively (P = 0.001); and for the control group, it was 37.6 ± 16.4 and 55.6 ± 14.5, respectively (P < 0.001). Also, there was a statistically significant difference in disability indices between the two groups after the intervention (P < 0.001).

**Conclusion:** Self-care program was suitable for needs assessment and provided basis for acquiring positive results in order to decrease disability and saved patient treatment costs.

## Introduction

Migraine is a common and disabling neurological disorder with a high prevalence in the first three decades of life.^1^^-^^3^ Repeated attacks of headache accompanied by nausea and vomiting lead to considerable disability, dysfunction, and lack of recreational and social activities, and inefficiency.^4^^-^^6^ Migraine induced disability is so severe that the World Health Organization (WHO) has listed it among the most disabling diseases.^7^^-^^9^ Given the unpredictable nature of migraine attacks and their negative effects such as dysfunction, quality of life issues, and family relationships, it could impose serious limitations on the individuals life.^6^^,^^7^^,^^9^^,^^10^ Migraine attacks not only influence the patients and their families but also impact the social and economic systems through direct and indirect disabilities.^11^^,^^12^ Studies have reported all the direct and indirect estimated expenses of migraine to be annually between 14 to 20 billion dollars, a major part of which is related to indirect expenses.^4^^,^^13^^-^^15^ Accordingly, the WHO in their global campaign has introduced measures for reducing the migraine-induced disability as an urgent priority of the public health in order to decrease the burden of migraine.^16^^,^^17^ Hence, considering the disabling consequences of migraine on social activities, family relationships, and its adverse social and economic consequences and WHO’s emphasis on reducing migraine-induced disability, it is important to consider self-care in these patients.^18^

One of the nursing models that is based on the ability of people in their self-care is "Orem’s self-care nursing model". Orem describes self-care as practical activities that the individuals perform in order to maintain life, health, and well-being.^19^^,^^20^ Those who are capable of self-care to satisfy the continuous requirements of maintaining life, health, and well-being are self-care agents. According to Orem, when the self-care agents are not able to satisfy self-care requirements on their own, they need nursing systems to sustain their health status.^21^ Orem’s self-care nursing model describes nursing systems in three categories: wholly compensatory, partly compensatory, and supportive educative.^22^^,^^23^ The supportive educative nursing system is applicable for patients with chronic diseases seeking to improve their self-care.^22^ Due to the chronic nature of migraine headaches, the active involvement of patients as the self-care agents in self-care activities has a prominent role in comprehensive treatment of migraine which ultimately compels them toward better control of headache symptoms and reducing the costs and disability.^24^

Therefore, providing a self-care program appropriate for patient needs in a supportive educative nursing system format could improve self-care in patients suffering from migraine and reduce migraine-induced disability. Therefore, this study was designed to determine the effectiveness of Orem's self-care program on headache related disability in migraine patients in Tehran, Iran.

## Materials and Methods

In this pre-post randomized clinical trial, 88 migraine patients admitted to neurology clinic in Baqiyaiatallah Hospital in Tehran were recruited. After obtaining approval from the ethics committee of the Baqiyatallah Medical Sciences University's Research Deputy, patients who signed the informed consent and met inclusion criteria were selected and randomly assigned to experimental or control groups with simple randomization method. The inclusion criteria included having episodic migraine headaches based on criteria of International Headache Society (with or without aura), aged 20-55 years, minimum ability of reading and writing, having no other disease or disability that affects quality of life such as psychological or other chronic diseases, no history of hospitalization due to headache, and patients who experienced at least five attacks in a month that attacks continued for 4-72 hours. The exclusion criteria included failure to perform the intervention properly, patients' unwillingness to continue participating in the study, and hospitalization due to migraine or other situation.

The instruments used for collecting data included a demographics questionnaire, Migraine Disability Assessment (MIDAS) questionnaire, Orem’s cognition form, and self-care checklist. MIDAS questionnaire is a standard questionnaire that its reliability has been confirmed in other studies by acceptable level of Spearman’s correlation coefficient (0.77-0.82).^25^^-^^27^ In this study, to determine the reliability of the MIDAS, daily diary card and test-retest were used. Spearman’s correlation coefficient test was 0.76 which confirmed its reliability. MIDAS is a short, self-administered questionnaire designed to quantify headache-related disability over a 3-month period. The scoring is based on five disability questions in three dimensions: two questions assess the number of missed or significant limitations to activity days (defined as at least 50% reduced productivity) due to headache in school or paid work activities (school/job dimension); two questions assess the number of missed or significant limitations to activity days (defined as at least 50% reduced productivity) due to headache in housework activities (housework dimension); one question assesses missed days due to headache in family, social, or leisure activities (social dimension). The total score is the sum of responses to questions 1-5. Two supplemental questions (A and B) provide the physician with additional clinical information about headache frequency and the average pain intensity (scale from zero to 10) of headaches over the previous three months. Based on the total scores, 4 disability grades are: 

Grade I (little or no disability, scores range 0-5), grade II (mild disability, scores range 6-10), grade III (moderate disability, scores range 11-20), and grade IV (severe disability, 21 or greater).^27^^-^^29^

At first participants of both groups completed the demographics and MIDAS questionnaire. Then, the control group received only usual treatment of the clinic. The intervention as well as usual treatment was performed for the experimental group as follows. First, Orem’s cognition forms were filled out by participant in order to determine self-care requirements and these needs were identified in terms of nutrition, physical activity, stress management, and sleeping improvement. Then, the self-care program was designed in three aspects of nutrition (following the diet properly), exercise (daily walking at least for 30 minutes), and progressive muscle relaxation (PMR) (for at least 20 minutes in the morning and at night) to control stress and improve sleep in the form of an Orem’s supportive educative nursing system. After the program design, the self-care program was taught in four theoretical and practical sessions of 30-45 minutes. The individual and group sessions were weekly held for patients in the experimental group for one month. The self-care checklist was provided to the patients at the end of each session (to follow up performance of the program) and patients were taught how to complete the checklist. Checklists were tabulated monthly and if the program was followed, the research unit checked the related option or put a negative sign if the program was not followed. At the end of the fourth session (last session of the theoretical training), the patients were provided with a training manual and requested to follow the self-care program and record their actions in the checklist for three months in order to reduce headache attacks and its disability. Meanwhile, the researcher evaluated performance of the intervention in the experimental group personally or by telephone and answered patients’ questions during the three months besides following up the intervention in the clinic. 

**Figure 1:**
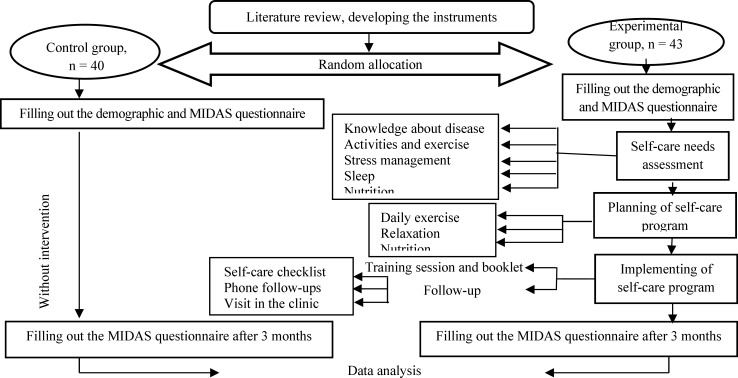
Study process

After three months, the self-care checklists of the experimental group were collected and the MIDAS questionnaire was completed personally by the experimental and the control group again. The study steps are shown in figure 1. 

MIDAS: Migraine Disability Assessment

Regarding the non-normal distribution, the data were analyzed using non-parametric tests by SPSS software (version 16, SPSS Inc., Chicago, IL, USA). The demographic variables were analyzed by chi-square, indicators of disability in patients with migraine were compared between groups by Mann-Whitney U test, and indicators of disability in patients with migraine were tested within groups by Wilcoxon test.

## Results

During the study, one patient in the experimental group and four patients in the control group were excluded due to their unwillingness to continue participation, and in total, 83 patients were studied. 

Most of the studied patients were female (73.5%) and aged between 20-30 years old. The chi-square test did not show any statistically significant difference between the two groups in terms of demographic variables (P > 0.050), which showed homogeneity of the groups (Table 1). 

Table 2 shows the mean disability index including the number of missed days and the days with reduced productivity due to migraine before and 3 months after intervention in the experimental and the control groups. Mann-Whitney test showed a statistically significant difference in disability indices of the experimental and control groups after the intervention (P < 0.001). 

The mean total MIDAS score in the experimental group was 28.1 ± 17.5, which corresponds to MIDAS grade IV (severe disability). However, after the intervention, the total MIDAS score in the experimental group decreased to 6.03 ± 4.52, which corresponds to MIDAS grade II disability. Wilcoxon test showed this difference was statistically significant (P = 0.001). But in the control group, the mean of total MIDAS score was 37.6 ± 16.4 before the study which corresponds to MIDAS grade IV; and at the end of study it increased to 55.6 ± 14.5, which is indicative of the increase in migraine-induced disability. The disability remained grade IV, and these statistics showed a statistically significant difference (P < 0.001).

Considering self-care needs before the intervention in the field of nutritional needs, 51.2% of patients in the experimental group had irregular nutrition program and 98.8% of them removed breakfast from their daily nutrition program. Also, 62.8% of them had no exercise practice; 55.8% had irregular sleep planning, 37.2% had moderate stress, and 20.9% suffered from severe stress. 

**Table 1:** Demographic characteristics of migraine patients in the two groups

**Demographic**	**Experimental** **Frequency (%)**	**Control** **Frequency (%)**	**P**
Age (year)			0.080
20-30	13 (30.2)	21 (52.5)	
31-40	11 (25.6)	11 (27.5)	
41-50	18 (41.9)	7 (17.5)	
> 50	1 (2.3)	1 (2.5)	
Sex			0.800
Female	31 (72.1)	30 (75.0)	
Male	12 (27.9)	10 (25.0)	
Education			0.620
Propaedeutic	6 (14.0)	3 (7.5)	
Diploma	22 (51.2)	21 (52.5)	
Bachelor’s degree or higher	15 (34.8)	16 (40.0)	
Marital status			0.170
Single	6 (14.0)	11 (27.5)	
Married	37 (86.0)	29 (72.5)	
Occupation			0.140
Homemaker	26 (60.5)	16 (40.0)	
Employee	11 (25.5)	13 (32.5)	
Other jobs	6 (14.0)	11 (27.5)	
Economy			0.760
Weak	2 (4.7)	2 (5.0)	
Moderate	33 (76.7)	28 (70.0)	
Good	8 (18.6)	10 (25.0)	

**Table 2:** Comparison of Migraine Disability Assessment (MIDAS) scores before and after the intervention in the two groups of patients with migraine

**MIDAS Subscale **	**Group**	**Experimental** **Mean ± SD**	**Control** **Mean ± SD**	**P**
Missed working days	Before	1.18 ± 2.18	1.47 ± 1.86	0.520
	After	0.00 ± 0.00	2.12 ± 2.75	0.001*
Days with reduced productivity	Before	5.04 ± 7.80	7.82 ± 7.88	0.110
	After	1.17 ± 2.67	11.10 ± 11.70	0.001*
Missed household work days	Before	5.72 ± 6.66	6.43 ± 4.17	0.560
	After	0.52 ± 1.32	10.40 ± 5.71	0.001*
Missed days of leisure activities, family and social	Before	4.59 ± 4.74	5.57 ± 3.77	0.390
	After	0.23 ± 0.47	6.57 ± 3.79	0.001*
Total MIDAS scores	Before	28.10 ± 17.5	37.60 ± 16.70	0.110
	After	6.03 ± 4.52	55.60 ± 14.50	0.001*

MIDAS: Migraine Disability Assessment; SD: Standard deviation

* P < 0.005

Regarding relaxation practices after the intervention (self-care checklists), 88.7% of the subjects in the experimental group performed relaxation exercises twice daily in the morning and at night; also 90.7% of them performed the daily aerobic exercise appropriately, and 88.4% of them properly adhered to self-care program in terms of nutrition.

## Discussion

The main finding of the present study was that Orem’s self-care program resulted in a significant decrease in headache-induced disability after the intervention in the experimental group compared to the controls. Investigating Orem’s self-care check list regarding nutrition, it was revealed that 88.4% of participants regularly adhered to self-care program regarding nutrition at home. Understanding the factors that trigger migraine attacks and changing or modifying the lifestyle are important factors in preventing migraine.^30^^,^^31^ It seems that the experimental group could better manage their headache attacks and reduce the disability by modifying their nutrition program, having their main meals, especially breakfast at regular intervals and avoiding foods that trigger migraines. Also, Buse et al.^32^ and Nazari and Eghbali^33^ in separate studies have greatly emphasized modifying nutrition patterns in order to prevent headache attacks and reduce disability.

In addition, results of the studies by Lemstra et al. in Canada^34^ and Smith et al. in Washington^18^ on the effect of a training intervention in patients with migraine revealed a significant decrease in number and severity of headache attacks after the intervention in the experimental group which is consistent with the results obtained from this study.

Stress and sleeping disorders are among other prevalent known triggers^35^^,^^36^ and in this study, the PMR was designed in order to reduce stress and improve the sleeping habits. Covering the whole body, PMR enables patients to concentrate on their body muscles and achieve complete relaxation.^10^ More than three-fourths of experimental group performed relaxation exercises in the morning and at night. Performing morning relaxation could lead to a day full of energy and exhilaration and performing night relaxation could lead to better sleeping. It is believed that when the body is relaxed, the mind cannot be in a state of panic and fear.^37^ Moreover, D'Souza et al.^38^ in their study aimed at the effect of relaxation training and written emotional disclosure on people with tension or migraine headaches showed that relaxation exercises compared to both written emotional disclosure and the control group improved headache and disability frequencies. Results of the studies during the period 2001 to 2009 revealed that relaxation significantly reduced stress, improved sleep, improved the mood of people suffering from migraine and consequently reduced headache and its consequent disability.^39^^-^^41^ These results all affirm findings of the present study.

Design of aerobic exercise programs in this study was performed based on the promising findings in various studies indicating the positive effects of aerobic exercises on reducing the frequency and severity of headache attacks and resulting disability.^42^^-^^44^ More than three-fourths of experimental group in this study performed the daily aerobic exercise properly. The main finding of the present study emphasizes the effect of exercise on reducing the number of headache attacks and the consequent disability. Varkey et al.^42^^,^^45^ revealed that performing aerobic exercises reduces the number, intensity and the duration of headache attacks. They concluded that exercising is an appropriate preventive option for migraine in patients who do not benefit from drug therapy or are not willing to take daily medicine.^31^^,^^42^^,^^45^ Also, similar results obtained in Koseoglu et al.^46^ study that showed that aerobic exercise increased plasma beta-endorphin level, and consequently increased pain threshold in patients with migraine and reduced number and severity of headache attacks. Results of the study by Dittrich et al.^40^ as well as the one carried out by Totzeck et al.^47^ also supported the findings of the present study regarding the positive effect of aerobic exercises on controlling and preventing the headache attacks and reducing the disability caused by headache. 

Moreover, results of the present study are consistent with the results acquired from the studies being conducted on self-care model format in accordance with the application of the self-care program in controlling the headache attacks and reducing the migraine disability. For example, Rosmawati et al.^22^ study about the evaluation of supportive-developmental nursing program on self-care practices of persons with type 2 diabetes showed that mean scores of total and subtotal self-care in the experimental group were significantly higher than those in the control group. Furthermore, Naji et al.^48^ study revealed a significant increase in the quality of all aspects of life in patients under hemodialysis in the experimental group after performing Orem’s self-care program compared to the control group. Baraz et al.^49^ study also showed that training the elderly by Orem’s self-care pattern increased the quality of all aspects of their life in comparison to the control group. Hamedanizadeh et al.^50^ study also revealed a significant decrease in headache indexes in the experimental group after performing Orem’s self-care program compared to the control group.

One of our findings was that in the control group, despite receiving medical treatment and proper medications, the severity of disability was increased at the end of study. The question that arises here is whether performed medical interventions or prescribed drugs did not work properly or the increase in the indicators was related to something else. Medication, especially new drugs to reduce headache attacks were largely successful so the cause cannot be attributed to lack of efficacy of drugs or medical procedures. According to literature, this phenomenon in the control group could be justified by non-compliance behaviors. Lehane and McCarthy^51^ in their study concluded that 30 to 50 percent of medications were not taken as prescribed and this led to adverse effect of drugs, adverse outcomes of disease, and increase in health care costs. Hekmatpour, et al.^52^ study showed that the main reasons for non-compliance are undesirable outcomes of initial treatment, frequent visits by multiple physicians with different experience, drug interactions, tiredness from taking the medication, and lack of patient education. The same reasons were seen in this study.

## Conclusion

According to the result, mean total scores of MIDAS in experimental group showed a significant decrease after intervention compared to the control group. Therefore, we found that self-care programs are suitable for the needs assessment, understanding the level of patients' information and self-care, and provide a basis for acquiring positive results in order to decrease disability and save patient treatment costs. On the other side, since migraine headaches have a chronic nature and most patients are not willing to take long term medication and considering migraine is prevalent in the first three decades of life, most patients have the required ability to perform the self-care and provide the life sustaining requirements. Consequently, self-care activities could be considered as an important part of comprehensive migraine treatment. Because migraine patients are treated as outpatient in pain clinics, it is appropriate to provide self-care programs suitable to their needs and based on patients' information and to provide a basis for acquiring positive results in order to decrease disability and save treatment costs.

## Notes:

**How to cite this article:** Mahmoudzadeh-Zarandi F, Hamedanizadeh F, Ebadi A, Raiesifar A. The effectiveness of Orem's self-care program on headache-related disability in migraine patients. Iran J Neurol 2016; 15(4): 240-7.
